# A G-type lectin receptor-like kinase TaSRLK confers wheat resistance to stripe rust by regulating the reactive oxygen species signaling pathway

**DOI:** 10.1007/s44154-025-00225-w

**Published:** 2025-05-23

**Authors:** Erbo Niu, Yibin Zhang, Henghao Xu, Bingliang Xu, Qiaolan Liang, Huixia Li, Jiahui Wang

**Affiliations:** College of Plant Protection, Gansu Agricultural University, Lanzhou, 730070 China

**Keywords:** Receptor like kinases, Peroxidase, Wheat stripe rust, Reactive oxygen species

## Abstract

Wheat stripe rust, caused by an obligate biotrophic pathogen *Puccinia striiformis* f. sp. *tritici* (*Pst*) seriously threatens wheat production. Discovering and utilizing of wheat resistance genes is the most effective and economical method to control diseases. The G-type lectin receptor-like kinase (LecRLKs) involved in biotic stress perception, while their roles in wheat resistance to *Pst* remain elusive. In our study, we identified 398 G-type LecRKs in wheat through BLAST and HMM profiling. The transcript level of 16 random selected G-type LecRKs from each subfamily were analyzed and found TaSRLK is highly induced by avirulent *Pst* CYR23 infection. TaSRLK-silenced wheat plants showed reduced resistance to *Pst* with increased hyphal length and decreased H_2_O_2_ accumulation. Surprisingly, TaSRLK was localized to the chloroplast and can induce cell death in *Nicotiana benthamiana*. Further, TaSRLK was shown to interact with and phosphorylate a peroxidase TaPrx1. Importantly, TaPrx1 involved in wheat resistance to *Pst* through regulating reactive oxygen species (ROS) production. Together these findings demonstrate that TaSRLK positively modulates ROS-associated wheat resistance by binding with TaPrx1.

**Supplementary Information:**

The online version contains supplementary material available at 10.1007/s44154-025-00225-w.

## Introduction

Upon perception of pathogens, plants activate a battery of immune system responses to defend pathogen infections, mainly including PAMP-triggered immunity (PTI) and effector-triggered immunity (ETI) (Shiu and Bleecker. 2001). Receptor-like kinases (RLKs) or receptor-like proteins act as pattern recognition receptors (PRRs) to detect pathogen-associated molecular patterns (PAMPs), subsequently trigger PTI (Wu and Zhou 2013). The leucine-rich receptor-like kinase (LRR-RLK) FLS2 (flagellin-sensitive 2) in Arabidopsis, one of the best-characterized PRRs, recognizes a bacterial flagellin protein (flg22) to trigger plant immune responses (Chinchilla et al. 2006; Zipfel et al. 2004). In rice, the *Xa21* gene codes for an LRR-RLK, containing 23 extracellular LRR repeats of 24 amino acids each and an intracellular protein kinase domain, which provides resistance to Xoo (*Xanthomonas oryzae pvoryzae*) by sensing and binding Ax21 (Song et al. 1995). The Arabidopsis chitin elicitor receptor kinase 1 (AtCERK1), a LysM-containing RLK, is crucial for activating the plant immune system (Liu et al. 2012). Conversely, successful pathogens produce effector proteins into plant cells to interfere with PTI and then facilitate infection. Plants evolved resistance proteins that recognize effector proteins derived from pathogens, thereby activating ETI (Ngou et al. 2022; Chisholm et al. 2006). Cysteine-rich receptor-like kinase 10, has been found interacts with the secreted effector CdE1 of *Coniella diplodiella*, and mediates resistance to *C. diplodiella* in grapevine (Liu et al. 2024). These findings indicate the crucial roles of RLKs in PTI and ETI.

Lectin RLKs constitute a subfamily of RLKs characterized by an extracellular lectin domain, a transmembrane (TM) domain and an intracellular kinase domain (Sun et al. 2020; Xu et al. 2023). Based on the variability of extracellular lectin domains, LecRKs are categorized into three groups: G-(B-lectin), C- and L-types (Sun et al. 2020; Teixeira et al. 2018; Xu et al. 2023). Among them, G-type LecRKs are proteins with an ectodomain similar to the mannose-binding motif of mannose agglutinin (Cheng et al. 2013) and also known as S-domain RLKs due to the presence of the S-locus region (Singh and Zimmerli 2013). Recent studies have highlighted the crucial roles of some G-type LecRKs in the plant immune system. For instance, the tobacco G-type LecRK ERK1 regulates the recognition of PcEXLX1 (a *Phytophthora capsica* expansin-like protein) and positively involved in resistance against *P. capsica* (Pi et al. 2022). NbLRK1, interacts with *Phytophthora infestans* INF1 elicitin and mediates INF1-induced cell death (Kanzaki et al. 2008). Additionally, in Arabidopsis, the binding of flg22 induces FLS2 heteromerization with BAK1 (Brassinosteroid receptor-associated kinase-1), triggering immune responses (Chinchilla et al. 2007). The rice Pi-d2, a G-type LecRKs with an S-locus region, provides resistance against the fungal pathogen *Magnaporthe grisea* (Chen et al. 2006). MdSRLK3 encodes an S-domain RLK, which isolated from apple and positively regulated apple resistance to *Valsa mali* (Han et al. 2022). Overall, G-type LecRKs play important roles in plant response to biotic stress.

Wheat stripe rust, caused by *Puccinia striiformis* f. sp. *tritici* (*Pst*), is one of the most destructive diseases of wheat worldwide, causing severe yield losses (Bhardwaj et al. 2019). Two strategies are available to control rust in wheat, including genetic resistance and chemical control. The most effective, economical, and environmentally friendly method is to identify and utilize resistant genes (Ellis et al. 2014). While with the rapid evolution of *Pst* new physiological races, wheat resistance provided by many race-specific resistance genes can be frequently overcome (Wang et al. 2016). For example, newly *Pst* races TSA-6 and TSA-9 have shown virulence to wheat *Yr5* gene, which has been used in wheat breeding programs (Goher et al. 2024; Zhang et al. 2020). Therefore, it is crucial for explore new genes that exhibit resistance to stripe rust. To date, the function of G-type LecRKs against infection by *Pst* in wheat is unknown. In our study, we identified 398 G-type LecRKs in wheat and found *TaSRLK* expression in Suwon was significantly induced upon *Pst* strain CYR23 infection. By contrast, *TaSRLK* expression showed no obvious changes in susceptible wheat cultivar MX 169 inoculated with *Pst*, suggesting that it could participated wheat resistance to *Pst*. TaSRLK-silencing plants showed suppressed wheat resistance to *Pst* as compared with the control plants with increased hyphal length and reduced H_2_O_2_ area. Unlike normal RLKs, TaSRLK was localized to the chloroplast and can induce cell death in *Nicotiana benthamiana*. Furthermore, TaSRLK was found to be interact with and phosphorylate a peroxidase protein named TaPrx1, which involved wheat resistance to *Pst* through affecting reactive oxygen species (ROS) production. In general, our study revealed that G-type LecRKs protein TaSRLK could bind with a peroxidase TaPrx1, and active wheat resistance against stripe rust by modulating ROS accumulation.

## Results

### Genome-wide identification and characterization of G-type LecRK family genes in wheat

**Fig. 1:**
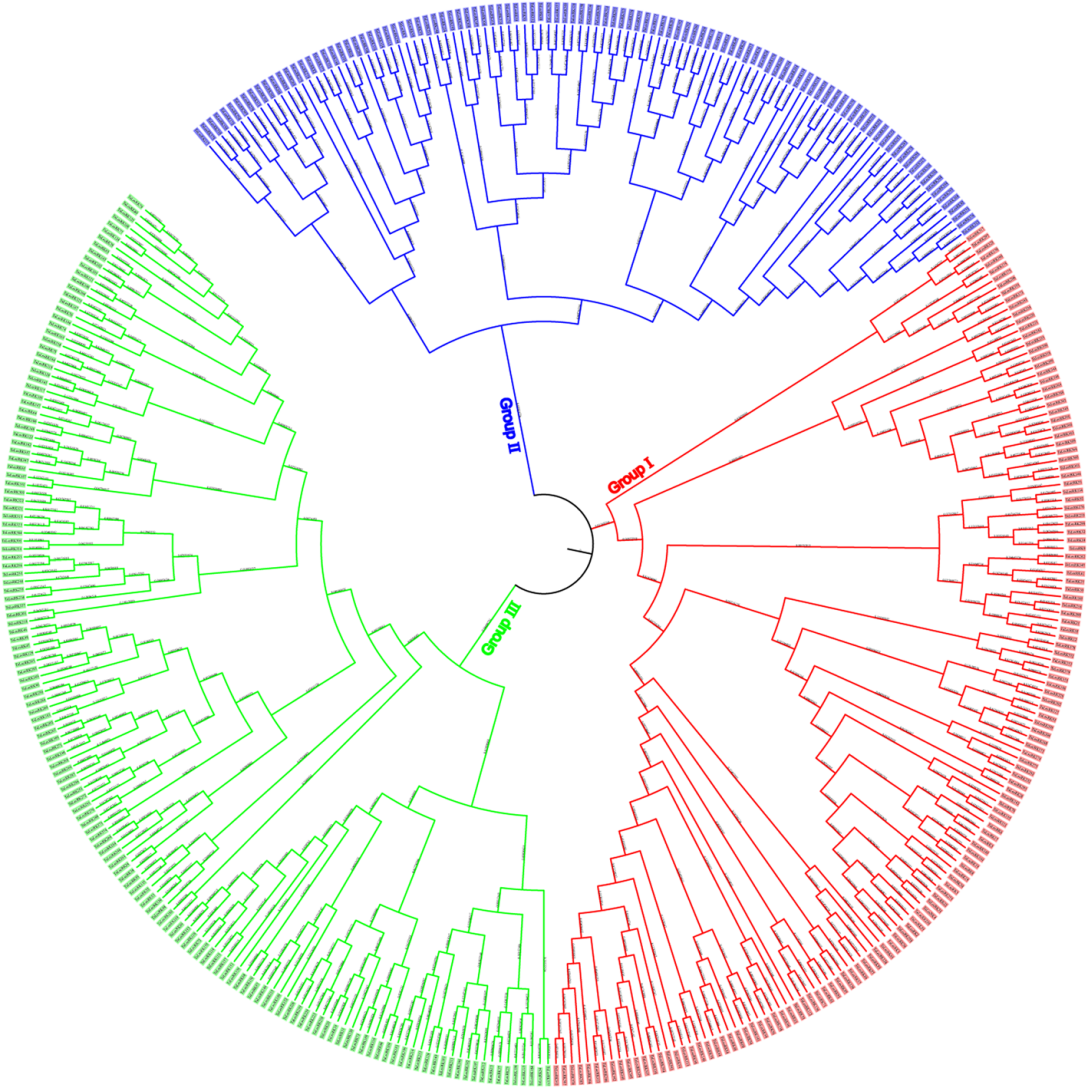
Phylogenetic tree for G-type TaLecRKs identified in *Triticum aestivum*. The amino acid sequences of G-type TaLecRKs from *T. aestivum* were aligned and the phylogenetic tree was constructed using MEGA 6.0 by the maximum likelihood method with 1000 bootstrap replicates

A total of 398 G-type LecRK proteins were identified from wheat genome and were divided into three major groups based on phylogenetic analysis. According to their positions on the chromosomes, we renamed them from TaLecRK1 to TaLecRK398. Group III the largest group contained 149 members, followed by group II and group I, which contained 142 and 107 members, respectively (Fig. 1).

### Expression patterns of G-type LecRKs in response to stripe rust

To determine the function of wheat G-type LecRKs in response to stripe rust, one G-type LecRK from each clade was randomly selected to analyze its respective expression profiles after *Pst* inoculation using qRT-PCR. Among 16 G-type TaLecRKs, *TaLecRK325* (later named TaSRLK) showed the highest up-regulation in wheat Suwon leaves inoculated with avirulent *Pst* CYR23 (Fig. S1). Whereas, *TaSRLK* expression showed no obvious changes in susceptible wheat cultivar MX 169 inoculated with *Pst* (Fig. S2). These findings suggest that TaSRLK might play a role in defense response against *Pst*.

### Silencing of *TaSRLK* significantly weakens wheat resistance against *Pst*

**Fig. 2:**
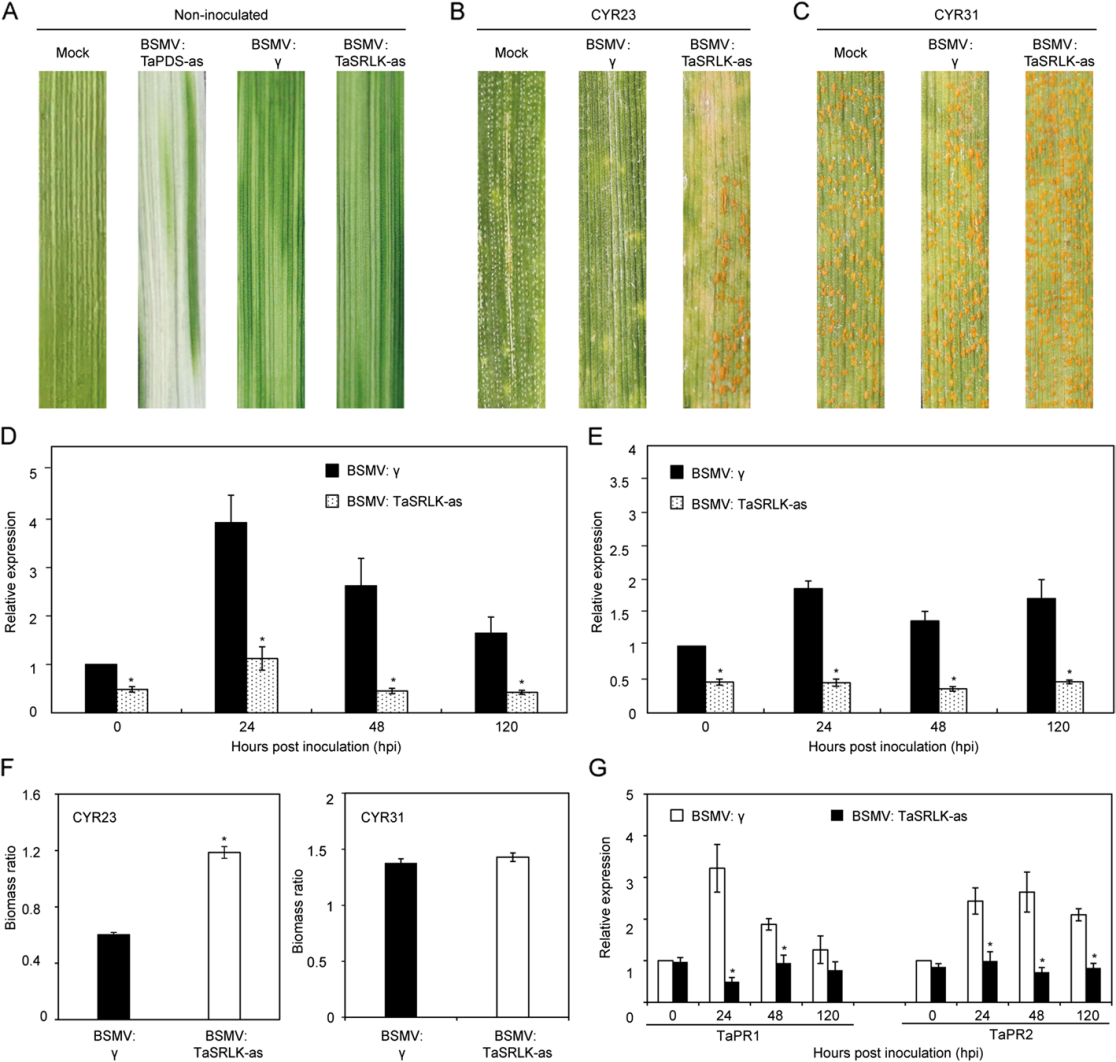
Silencing *TaSRLK* decreases wheat resistance to *Pst* race CYR23. **A** Symptom of wheat leaves infected with BSMV:00, BSMV:TaPDS or BSMV:TaSRLK. Mock wheat leaves were inoculated with 1 × FES buffer as a control. Disease symptom of TaSRLK-silenced and non-silenced leaves infected with avirulent CYR23 (**B**) or virulent CYR31 (**C**). The expression levels of *TaSRLK* in TaSRLK-silenced wheat leaves during the interaction with CYR23 (**D**) or CYR31 (**E**) *Pst* races. Asterisks indicate the presence of statistically significant between BSMV:γ and BSMV: TaSRLK-infected leaves at each time point (*P < 0.05). **F** The biomass ratio (*Pst*/wheat) in non-silenced and TaSRLK-silenced wheat leaves inoculated with CYR23 or CYR31 at 14 dpi (days post inoculation). Means and standard deviations were calculated from three independent replicates. **G** The *PR* gene expression in TaSRLK-silenced leaves inoculated with CYR23. The transcript level of *PR* genes in non-silenced plants at 0 h post inoculation (hpi) was standardized as 1. Asterisks indicate significant differences at the same time points between BSMV:γ and BSMV:TaSRLK by Student’s *t*-test (*P < 0.05)

To investigate the function of *TaSRLK*, we utilized a VIGS system to generating TaSRLK-silenced wheat plants. Plants infected with BSMV showed symptoms of chlorotic mosaic at 10 days post-inoculation (dpi), and leaves infected with BSMV: TaPDS exhibited photobleaching, validating the efficiency of the BSMV-induced gene silencing system (Fig. 2A). At 14 days after inoculation with *Pst* CYR23, wheat leaves showed disease resistance to *Pst*, whereas TaSRLK-silenced leaves exhibited decreased resistance with a few fungal uredia (Fig. 2B). In contrast, all leaves inoculated with *Pst* CYR31 showed susceptible symptoms (Fig. 2C). The expression level of *TaSRLK* in TaSRLK-silenced plants was significantly lower than that in non-silencing plants during wheat and *Pst* interaction, suggesting *TaSRLK* was suppressed successfully through BSMV system (Fig. 2D-E). The *Pst* biomass was also increased in the TaSRLK-silenced wheat leaves inoculated with CYR23 (Fig. 2F). Moreover, we found *TaPR1* and *TaPR2* were decreased in TaSRLK-silenced plants infected with CYR23 (Fig. 2G).

**Fig. 3:**
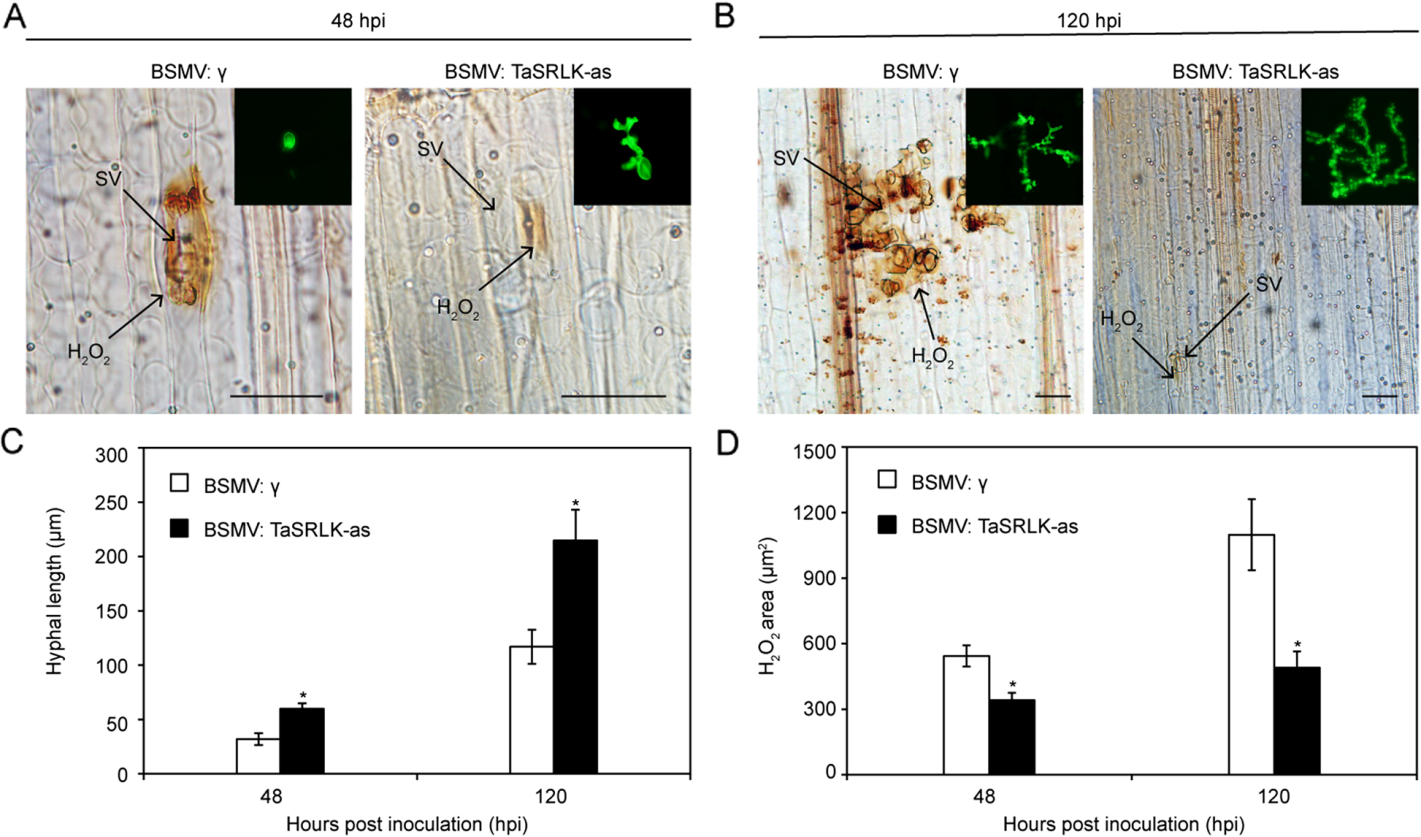
Silencing *TaSRLK* significantly increases *Pst* hyphal expansion and reduces H_2_O_2_ accumulation. Microscopy observation of fungal growth and host responses in BSMV-infected leaves at 48 (A, bar = 20 µm) and 120 h post inoculation (hpi) (B, bar = 50 µm) with *Pst* race CYR23. SV, substomatal vesicle. Quantification of hyphal length and H_2_O_2_ area in BSMV-treated wheat plants inoculated with virulent *Pst* race CYR23 at 48 and 120 hpi. The hyphal length represents the average distance (μm) measured from the junction of the substomatal vesicle to the hyphal tip. The results were obtained from three biological replicates with 30 infection sites per replicates. The means and standard deviations from three independent replicates were calculated, and significant differences (*P < 0.05) were denoted by one asterisk (Student’s *t*-test)

In addition, histological analysis were observed in wheat leaves inoculated with *Pst* CYR23 based on staining with 3,3'-diaminobenzidine (DAB) and wheat germ agglutinin (WGA). As shown in Fig. 3A-D, TaSRLK-silenced leaves showed an enhanced *Pst* extension with significantly increased hyphal length and reduced H_2_O_2_ area in comparison with non-silenced leaves. These results confirmed that silencing *TaSRLK* weakens wheat disease resistance to *Pst*.

### The chloroplast protein TaSRLK induced cell death in tobacco

**Fig. 4:**
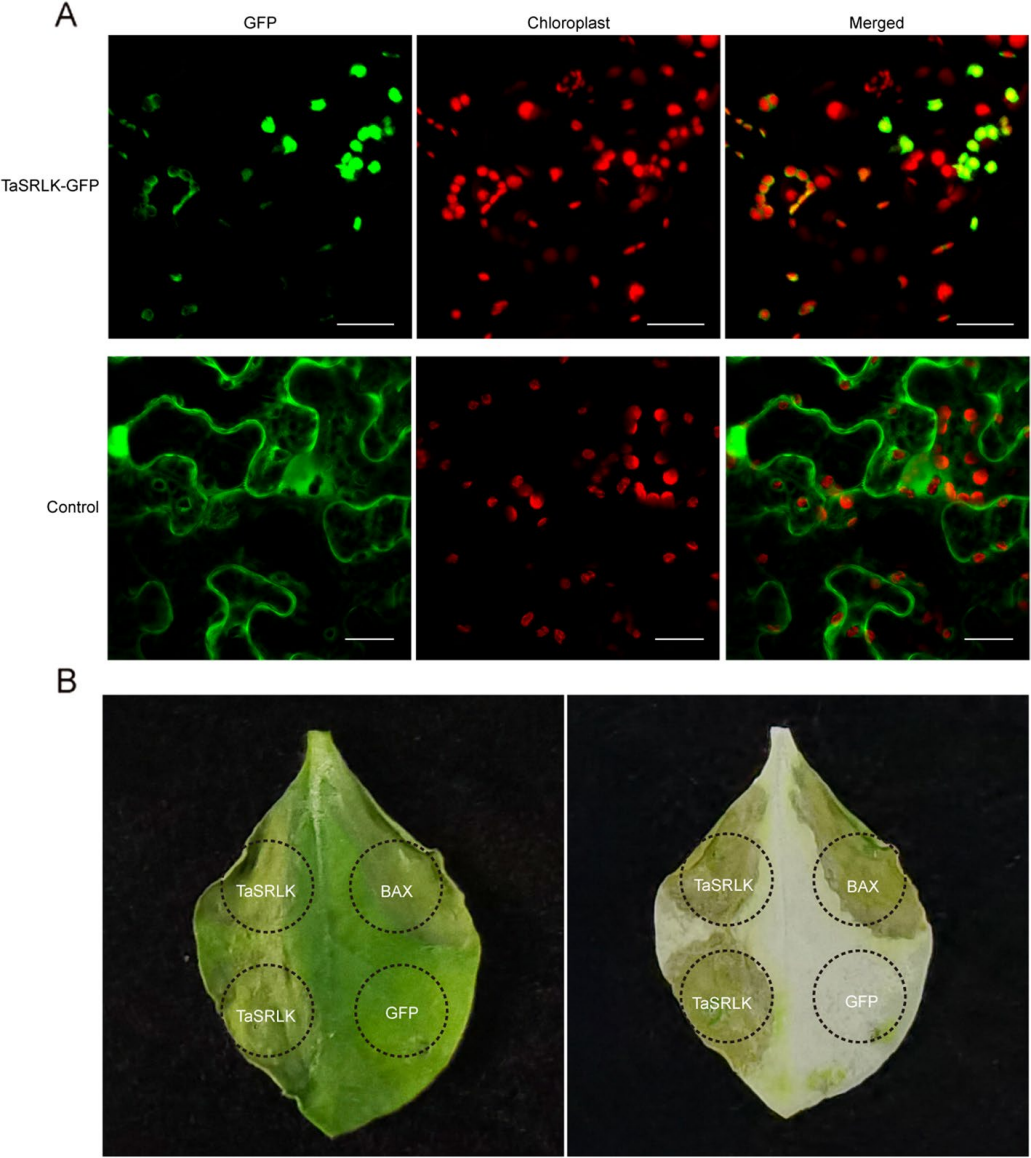
Transient expression of *TaSRLK* in *Nicotiana benthamiana* leaves. **A** Subcellular localization of TaSRLK. Green fluorescence indicates GFP signal, red fluorescence indicates chloroplast autofluorescence. Bar = 20 μm. **B** Phenotypes of *N. benthamiana* leaves overexpressing* TaSRLK*

To identify the subcellular localization of TaSRLK, the TaSRLK-GFP fusion protein was introduced into *N. benthamiana* leaves via agroinfiltration. Consistent with the bioinformatics analysis results, GFP from the TaSRLK fusion constructs was observed predominantly in the chloroplast (overlapped with red fluorescence produced by the chloroplast autofluorescence), whereas the control GFP showed widespread distribution throughout the whole cell (Fig. 4A).

To investigate the role of TaSRLK in cell death, the coding region of TaSRLK was transiently expressed in *N. benthamiana* leaves through potato virus X (PVX) delivery. Similar to the positive control (proapoptotic protein BAX), expression of TaSRLK in *N. benthamiana* leaves induced cell death. Conversely, no symptoms were detectable for the negative control (PVX-GFP) (Fig. 4B).

### TaSRLK interacts with and phosphorylates TaPrx1

**Fig. 5:**
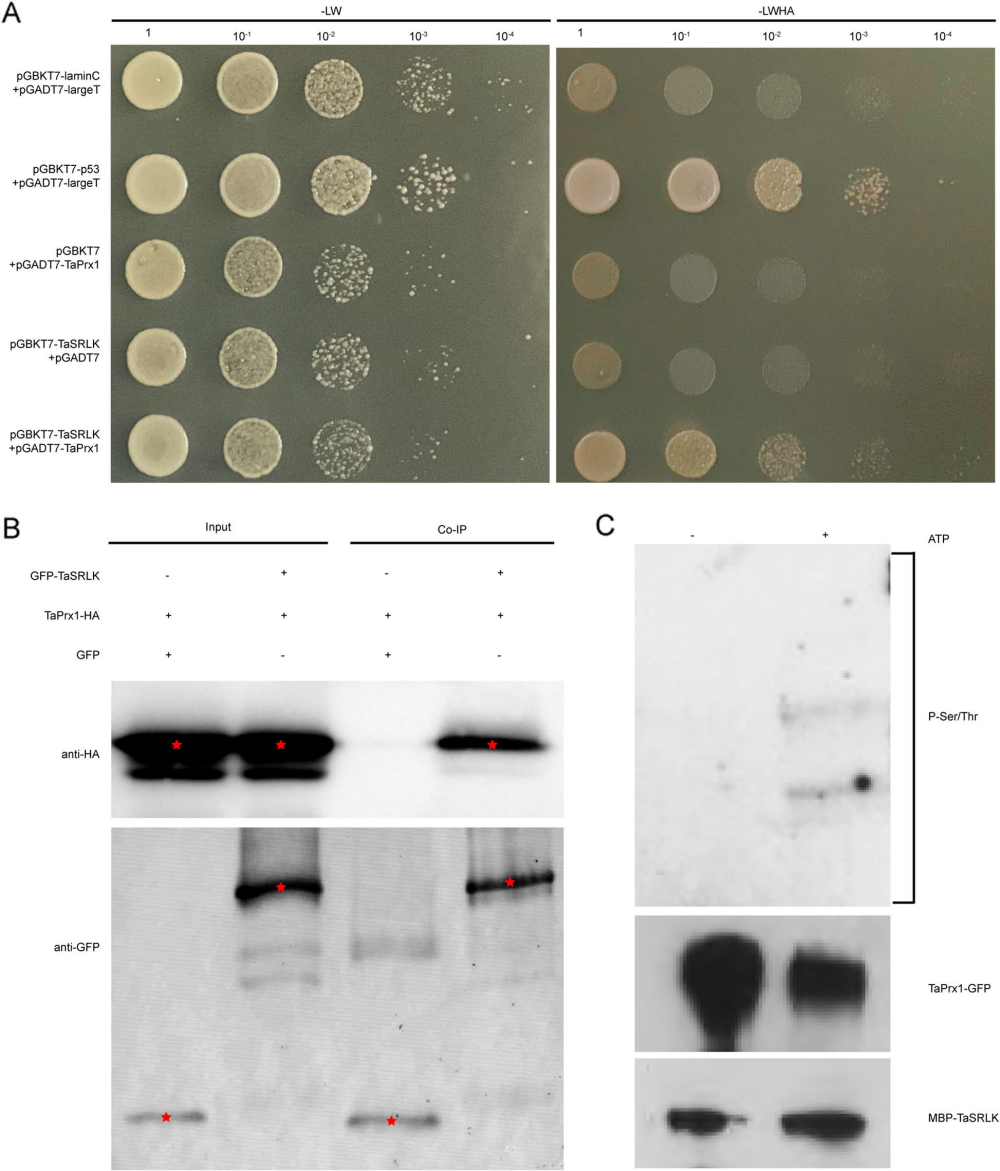
TaSRLK interacts with and phosphorylates TaPrx1. **A** Yeast two-hybrid of TaSRLK and TaPrx1 interaction. Yeast cells transformed with constructs were grown on -LW (lacking leucine and tryptophan) and -LWHA (lacking leucine, tryptophan, histidine and adenine). **B** Co-immunoprecipitation assay showing the TaSRLK-TaPrx1 interaction. Co-overexpression of pBin61-GFP-TaSRLK and pBin41-TaPrx1-HA in *Nicotiana benthamiana* leaves. The coprecipitation was analyzed by western blot using anti-HA and anti-GFP antibody. **C** Phosphorylation of TaPrx1 by TaSRLK in vitro. MBP-TaSRLK and TaPrx1-GFP proteins were incubated in the presence or absence of ATP. The phosphorylated proteins were analysed by western blot

To gain deeper insights into the role of TaSRLK in wheat immune response to *Pst*, we utilized this protein as a bait in yeast two-hybrid screening using the Suwon11 cDNA library to identify its interacting proteins. By the two rounds of screening performed, we identified a putative TaSRLK-interacting protein peroxisome1 like (TaPrx1). To further validate the interaction between TaSRLK and TaPrx1, Y2H and co-immunoprecipitation (Co-IP) assays were performed. In Y2H experiment, except for the positive control, normally grown yeast on SD/-Trp/-Leu/-His/-Ade medium was observed only in the presence of both BD-TaSRLK and AD-TaPrx1 (Fig. 5A). Co-IP results showed that TaSRLK-GFP, but not the empty vector (EV) GFP, could interact with TaPrx1-HA in vivo. These results suggest that TaSRLK interacts with TaPrx1 (Fig. 5B). In addition, the results of in vitro phosphorylation assays showed that TaSRLK phosphorylates TaPrx1 (Fig. 5C).

### Knockdown of *TaPrx1* compromised wheat resistance to an avirulent strain of stripe rust

**Fig. 6:**
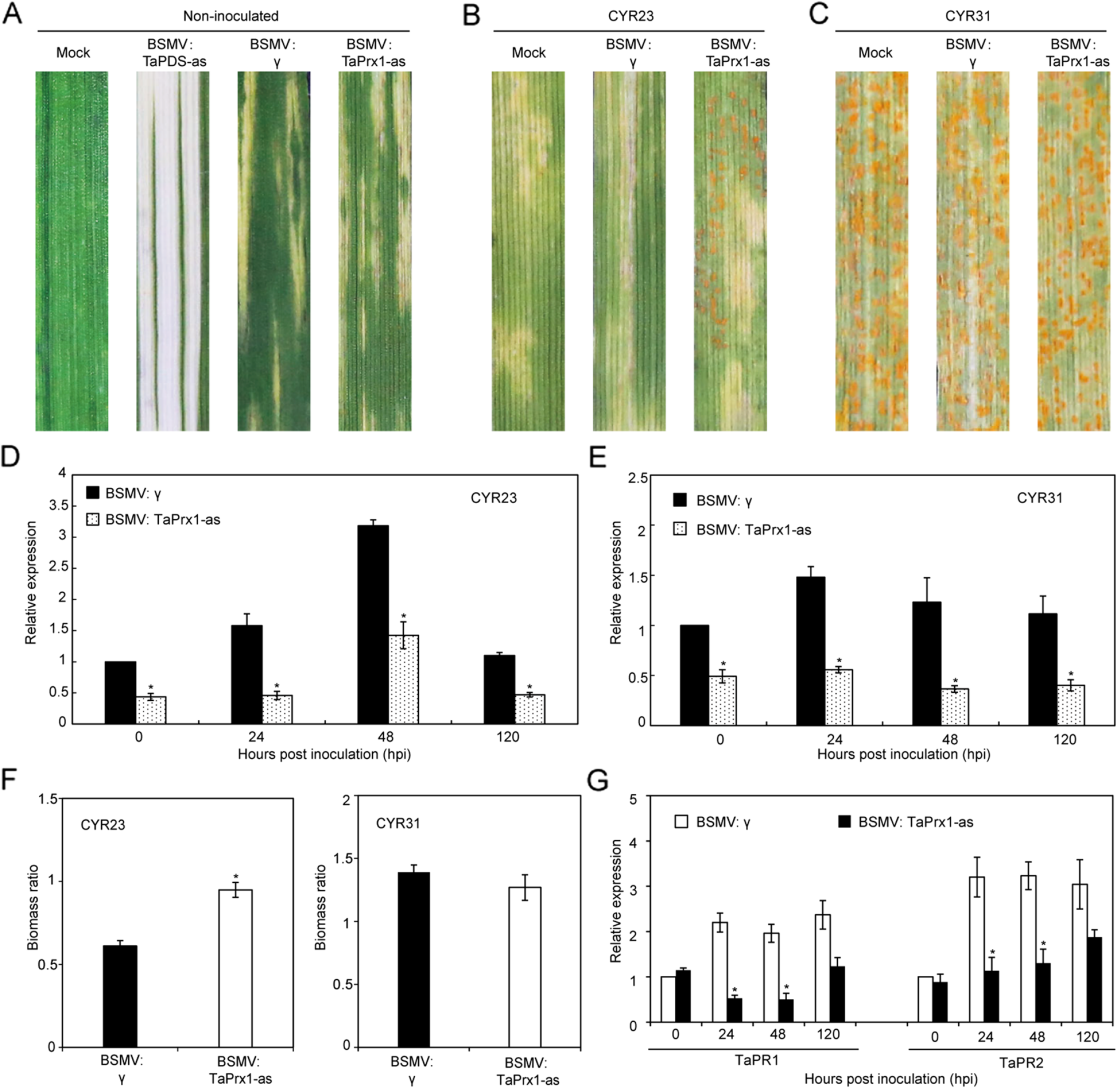
Reducing the expression of *TaPrx1* compromises wheat resistance to *Pst* race CYR23. **A** Phenotypes of BSMV-treated wheat leaves. Mock: wheat leaves treated with 1 × FES buffer. Disease phenotypes of the fourth leaves pre-inoculated with BSMV and then challenged with the avirulent CYR23 (**B**) or the virulent CYR31 (**C**). The expression levels of *TaPrx1* during the interaction with *Pst* races CYR23 (**D**) or CYR31 (**E**) in BSMV-treated wheat plants. The error bars reflect variations among three independent replicates. Asterisks indicate significant differences between BSMV:γ and BSMV:TaPrx1 by Student’s *t*-test (*P < 0.05). **F** The biomass ratio (*Pst*/wheat) in non-silenced and TaPrx1-silenced wheat leaves at 14 dpi (days post inoculation). Means and standard deviations were calculated from three independent replicates. **G** Transcripts of defense-related genes *TaPR1* and *TaPR2* in TaPrx1-silenced plants. The asterisks mark significant differences by Student’s *t*-test (*P < 0.05)

To explore whether *TaPrx1* involved in wheat resistance to *Pst*, we silenced *TaPrx1* by BSMV-induced VIGS and evaluated this impact on wheat immune response to *Pst* infection. After BSMV-inoculated wheat leaves displayed exhibited typical BSMV infection symptoms (Fig. 6A), the fourth leaves were inoculated with *Pst* isolate CYR23 or CYR31. By 14 dpi, the TaPrx1-silenced wheat plants appeared large numbers of uredia compared with non-silenced plants infected with CYR23 (Fig. 6B). By contrast, there was no obvious phenotypic difference between BSMV:γ and BSMV:TaPrx1 in the compatible interaction, which produced numerous uredia on all leaves inoculated with CYR31 (Fig. 6C). The transcript levels of the *TaPrx1* genes was reduced more than 50% at 0, 24, 48, and 120 hpi in BSMV:TaPrx1 infected plants (Fig. 6D-E). The biomass analysis showed that the biomass of *Pst* in TaPrx1-silenced wheat plants inoculated with CYR23 was significantly increased (Fig. 6F). In addition, two PR genes (*TaPR1* and *TaPR2*) showed lower expression levels than that in the non-silenced leaves (Fig. 6G).

**Fig. 7:**
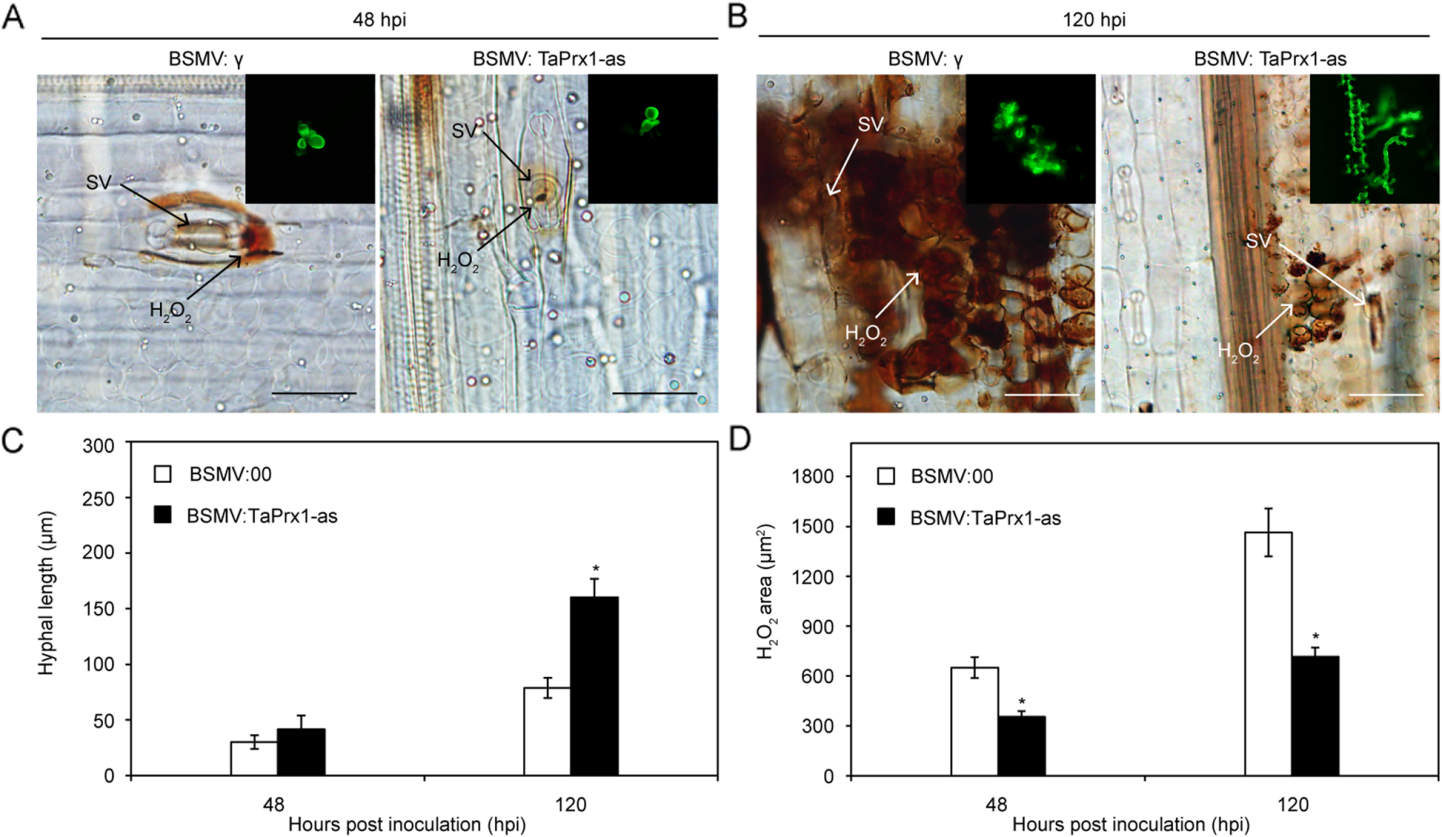
Histological observations of *Pst* growth and wheat responses in leaves inoculated with BSMV: γ or BSMV:TaPrx1 and infected with *Pst* race CYR23. Fungal growth and H_2_O_2_ accumulation in BSMV-treated leaves at 48 (A, bar = 20 µm) and 120 h post inoculation (hpi) (B, bar = 50 µm) with CYR23. SV represents for substomatal vesicle. A statistical histological analysis of hyphal length and H_2_O_2_ area in TaPrx1-silenced leaves inoculated with CYR23 at 48 and 120 hpi. The hyphal length is the average distance (μm) from the junction of the substomatal vesicle to the hyphal tip. Three independent biological replicates with 30 infection sites per replicates were performed. The mean and standard deviation were calculated from three independent replicates. Asterisks indicate significant difference (*P < 0.05, Student’s *t*-test)

Histological analysis in TaPrx1-silenced plants infected with CYR23 were observed by microscopy (Fig. 7A-B). The hyphal lengths was significantly increased in the TaPrx1-silenced plants at 120 hpi (Fig. 7C), and H_2_O_2_ area was significantly reduced in the TaPrx1-silenced plants at 48 hpi and 120 hpi (Fig. 7D). Our results clearly illustrated that *TaPrx1* positively regulates wheat resistance against the avirulent *Pst* CYR23.

## Discussion

G-type LecRKs, which belongs to the sub-family of RLKs, play a crucial role in both biotic and abiotic stress responses (Kim et al. 2009; Bellande et al. 2017; Franck et al. 2018; Xu et al. 2023). However, few studies have reported the deeply molecular mechanism in G-type LecRKs-regulated plant defense immunity. PWL1 encodes a G-type LecRK with an extracellular signal peptide (SP) domain, a classic bulb-lectin domain (B-lectin) domain, an S-locus glycoprotein (SLG) domain, a plasminogen-applenematode (PAN) domain, a TM domain and an intracellular serine/threonine kinase (STK) domain. Mutation of *PWL1* showed markedly enhanced disease resistance to bacterial pathogens *Xoo* and Xoc by promoting the accumulation of ROS, salicylic acid (SA) and jasmonic acid (JA) (Xu et al. 2023), however, the mechanism by which PWL1 affects ROS production has not been elucidated. In our study, we identified a typical G-type LecRK named TaSRLK that rapidly induced by *Pst* infection. Silencing of *TaSRLK* by VIGS exhibited significantly reduced ROS bursts and wheat resistance to *Pst*. In addition, we found TaSRLK can affect ROS production through interacting with peroxidase TaPrx1, thereby regulates wheat resistance response to *Pst*. Comparatively, in rice, a G-type LecRK SDS2 positively regulates rice resistance to blast fungus *Magnaporthe oryzae* through interacting with OsRLCK118/176, which phosphorylates the NADPH oxidase OsRbohB to induce ROS production (Fan et al. 2018). Overall, our study provides insights into the G-type LecRK-regulated wheat resistance molecular mechanism to *Pst*.

Plant peroxidases, an essential element of the plant antioxidant system, have multiple functions and serve a key regulatory role in responding to various stresses via generating or scavenging ROS (Kim et al. 2010; Torres 2010). Previous research has reported that upon pathogen infection, poplar peroxidase *PdePrx12* expression is suppressed, subsequently leading to increased H_2_O_2_ levels. In addition, *PdePrx12*-overexpression lines had a reduced H_2_O_2_ content, whereas *PdePrx12* reduced-expression lines had an increased H_2_O_2_ content (Cai et al. 2023). The ROS-scavenging activity of ascorbate peroxidase NbAPX3-1 was vital for its immune function. While the interaction between conserved RXLR effector PpE18 of *Phytophthora parasitica* and NbAPX3-1 lead to hinderance of ROS scavenging capabilities, increased ROS concentrations in peroxisomes and reduced *N. benthamiana* resistance (Cao et al. 2024). By contrast, CsPrx25-transgenic citrus plants induce resistance to *Xanthomonas citri* subsp. *citri* (*Xcc*) with enhanced H_2_O_2_ levels (Li et al. 2020). In Arabidopsis, peroxidase PRX33 and PRX34 knockdown lines exhibit educed ROS production in response to fungal and bacterial pathogens (Daudi et al. 2012). In our research, we found that silencing peroxidase *TaPrx1 *led to a decrease in H_2_O_2_ levels in wheat, suggesting TaPrx1 may regulate H_2_O_2_ generation.

Due to functional diversity of peroxidase, different studies have demonstrated different links between peroxidase and plant resistance. The *PdePrx12* gene negatively regulates poplar disease resistance to pathogens (Cai et al. 2023). Overexpression of *CaPO2* in Arabidopsis enhanced resistance to the hemibiotrophic bacterial pathogen *Pseudomonas syringae* pv. *tomato* DC3000 and necrotrophic fungal pathogen *Alternaria brassicicola* infection (Choi et al. 2007). *HvPrx40* and *TaPrx103* positively regulated wheat resistance to powdery mildew (Johrde and Schweizer 2008; Li et al. 2020). In our research, we identified a TaSRLK-interacting protein peroxidase TaPrx1. Silencing of *TaPrx1* result in reduced wheat resistance to avirulent *Pst* race CYR23 with increased hyphal length and decreased H_2_O_2_ area, indicating *TaPrx1* plays a positive role in wheat resistance. Overall, our study provides insights into the molecular mechanism of peroxidase in wheat resistance to *Pst*.

## Conclusion

In summary, G-type RLK TaSRLK could be quickly induced upon *Pst* infection and positively regulated wheat resistance to avirulent CYR23. Additionally, we identified a peroxidase TaPrx1 that was shown to interact with and phosphorylate TaSRLK. Silencing of *TaPrx1* reduced wheat resistance to *Pst* through affecting production of H_2_O_2_. Overall, chloroplast protein TaSRLK participates in the control of ROS production via interaction with TaPrx1, thereby enhanced wheat resistance to stripe rust. Our discoveries offer valuable insights into the molecular mechanisms by which TaSRLK regulates the resistance of wheat to *Pst*.

## Materials and methods

### Identification of G-type LecRKs family genes

The complete genome data and protein sequences of *Triticum aestivum* were downloaded from IWGSC resources (http://www.wheatgenome.org/, http://wheat-urgi.versailles.inra.fr/Seq-Repository/Genes-annotations). To identify wheat G-type LecRKs protein family, we utilized the Arabidopsis G-type LecRKs and rice G-type LecRKs sequences to perform BLAST searches. Proteins domains containing protein kinase domain (PF00069) and B-lectin (PF01453) were obtained from the Pfam database (https://pfam.janelia.org/) using the Hidden Markov Model (HMM) profile. We then use online tools SMART (http://smart.embl-heidelberg.de/) (Letunic et al. 2021) and NCBI-CDD (https://www.ncbi.nlm.nih.gov/cdd/) (Lu et al. 2020) to confirm the final G-type LecRKs. These final wheat G-type LecRKs genes were renamed based on chromosomal location. The subcellular localization information of the G-lectin RLK protein sequences were analyzed by the online website WoLF PSORT (http://www.genscript.com/wolf-psort.html) (Horton et al. 2007).

### Multiple sequence alignment and phylogenetic analysis

The protein sequences encoded by the wheat G-type LecRKs genes were used to conduct the phylogenetic tree analysis. MEGA 7.0 software was used to construct the phylogenetic tree by using the maximum likelihood (ML) method (1000 bootstrap replicates).

### Plant and fungal material

Wheat (*Triticum aestivum* L.) cultivar Suwon11, MingXian169 (MX169) and two stripe rust *Pst* races, CYR23 and CYR31 were used in this study. Seedlings of Suwon11 and MX169 (susceptible wheat cultivar) were grown, maintained and inoculated with the *Pst* pathotypes as described by Kang and Li (1984). Suwon11 contains the *YrSu* resistant gene, expresses resistance to CYR23 but susceptible to CYR31 (Cao et al. 2003).

### RNA Extraction and qRT-PCR

The SV Total RNA Isolation System (Promega, Madison, WI) was used to extract total RNA from wheat leaves according to the manufacturer’s instructions. Next, the PrimeScript®RT reagent Kit (TaKaRa, Tokyo, Japan) was employed to synthesize first-strand cDNA. The gene *TaEF-1α* (*T. aestivum* elongation factor 1 alpha, accession number: Q03033) was then normalized. QRT-PCR analysis was conducted with UltraSYBR One Step RT-qPCR Kit (CWBIO, Beijing, China). The quantitative real-time PCR data were evaluated utilizing the comparative 2 ^–ΔΔCT^ technique.

### Cloning of *TaSRLK* and sequence analysis

To obtain the full-length CDS region of *TaSRLK*, specific primers (forward and reverse) were designed based on International Wheat Genomic Sequence Consortium database (http://www.wheatgenome.org). The template was a mixture of RNA extracted from leaves of Suwon11 at 12, 24, 48, 72 h and 120 h post inoculated with CYR23. The resulting PCR products were cloned into the pGEM-T Easy vector (Promega, Madison, WI) and sequenced. TaSRLK amino acid sequences and the deduced protein sequences were analyzed using DNAMAN software v6.0 (Lynnon BioSoft, Quebec, Canada).

### BSMV-mediated *TaSRLK* gene silencing

BSMV RNAs were carried out as described by Wang et al. (2012). The second leaves of wheat seedlings were inoculated with each of the five viruses (BSMV:α, BSMV:β, BSMV:γ, BSMV:TaPDS and BSMV:TaSRLK) as following a previously published method by Scofield et al. (2005). Mock-treated plants were treated with 1 × FES buffer (0.1 M glycine, 0.06 M K_2_HPO4, 1% w/v tetrasodium pyrophosphate, 1% w/v bentonite, and 1% w/v celite, pH 8.5) as a control. The BSMV-infected wheat seedlings were kept in a growth chamber at 25 °C. Photobleaching symptoms were observed on BSMV:PDS seedlings after inoculation approximately 10–14 days. The fourth leaves in wheat then were inoculated with *Pst* (CYR23 or CYR31). Next, these plants were maintained at 16 °C and the fourth leaves sampled at 0, 24, 48 and 120 hpi for histological observation and RNA isolation. QRT-PCR analyses were conducted to confirm the silencing efficiency of *TaSRLK* for each assay. After 14 days, when extensive fungal growth was visible in leaves, infection phenotypes of *Pst* were recorded and photographed. For fungal biomass analysis, the relative quantification of the *Pst* gene *PstEF1α* and the wheat gene *TaEF-1α* (Panwar et al. 2013) were assessed to quantify the *Pst* and wheat DNA, respectively.

### Histological observation of fungal growth and ROS accumulation

Leaves were collected from *Pst*-infected plants at different time points (0, 24, 48, and 120 hpi) for DAB staining to detect ROS accumulation under an Olympus BX-51 fluorescence microscope (Olympus Corp., Tokyo, Japan). Applying wheat germ agglutinin (WGA) staining to leaf sections to monitor hyphal growth, as outlined by Wang et al. (2017). A total of thirty infection sites were examined on five randomly selected leaf segment for each treatment. Three independent biological repeats were conducted.

### Transient expression of proteins in plants

For sub-localization of TaSRLK, plasmids of TaSRLK-GFP or green fluorescent protein (GFP) were briefly expressed in the *Nicotiana benthamiana* leaves through agroinfiltration with a syringe lacking a needle. GFP fluorescence signals were observed and photographed at 48 h after infiltration.

For cell death analysis, the *N. benthamiana* leaves were observed and photographed after 72 h infiltration with agrobacterium containing PVX-TaSRLK, PVX-BAX (positive control) or PVX-GFP (negative control).

### Yeast two-hybrid (Y2H) assay

The complete TaSRLK coding sequence were inserted into the pGBKT79 (BD) vector. Then, pGBKT79 (BD)-TaSRLK and pGADT7-cDNA libraries were co-transformed into yeast strain AH109 according to the Yeast Protocols Handbook (Clontech). Transformants grown on SD/-Leu-Trp (SD/-LT) medium were cultured on SD-Leu-Trp-His-Ade containing X-a-gal to identify any possible interactions. For interaction analysis of TaSRLK and TaPrx1, the BD-TaSRLK and AD-TaPrx1 were co-transformed into yeast strain AH109 and finally plated on SD/-Leu/-Trp/-His/-Ade.

### Co-immunoprecipitation (Co-IP assay)

The *Agrobacterium tumefaciens* GV3101 strains containing pBin61-GFP-TaSRLK and pBin41-TaPrx1-HA, pBin61-GFP-TaSRLK and pBin41-HA or pBin61-GFP and pBin41-TaPrx1-HA vector combinations were transformed into *Nicotiana benthamiana* plants. After 60 h of infiltration, *N. benthamiana* leaves were collected for total protein extraction. Leaf samples were ground into powder in liquid nitrogen, and lysed by extraction buffer (10 mM Tris–HCl, pH 7.5, 150 mM NaCl and 0.5 mM EDTA, pH 8.0, 0.2% NP-40 and protease inhibitor cocktail; Roche, Branchburg, NJ, USA). Total proteins were then incubated with 40 µl GFP-Trap agarose beads (ChromoTek, Planegg-Martinsried, Germany) for 2 h at 4 ℃. The immunoprecipitated proteins were washed three times with washing buffer and boiled for 10 min in SDS protein loading buffer. The eluted proteins were subsequently analyzed by western blot using anti-HA antibody (Abcam, Cambridge, MA, USA) and anti-GFP antibody (CWBIO, Beijing, China).

### In vitro phosphorylation assay

In vitro phosphorylation assay was performed as described previously (Wang et al. 2021). Briefly, MBP-TaSRLK was expressed in *Escherichia coli* BL21 (DE3). TaPrx1-GFP was purified by GFP-Trap agarose beads. MBP-TaSRLK and TaPrx1-GFP complex in kinase buffer (Tris–HCl, pH 7.5, 1 M MgCl_2_, 1 M DTT) were incubated at 30 °C for 30 min with or without 25 mM ATP. After adding 5 × SDS loading buffer and boiled at 95 °C for 15 min, the phosphorylated proteins were detected with pan phospho-Serine threonine rabbit polyclonal antibody (Abcam, Cambridge, MA, USA).

### Statistical analysis

Each experiment involved at least three independent biological replicates. Mean values and standard errors were calculated using Microsoft Excel software. For statistical analysis, SAS (SAS Institute Inc., Cary, NC, USA) software was utilized (*P < 0.05).

## Supplementary Information

Supplementary Material 1: Figure S1 Expression pattern of 16 G-type *TaLecRKs* in response to *Pst* infection. Suwon11 leaves were inoculated with *Pst* race CYR23. RNA was extracted from the sampled leaves at 0, 12, 24, 48, 72 and 120 hours post inoculation (hpi). The 2^−ΔΔCT^ method was used for relative expression analysis. Wheat* TaEF-1α *was used as internal controls for normalization. To facilitate comparisons, the transcript levels of each *TaLecRK* at 0 hpi with CYR23 were set to 1. The error bars in the data represent the standard error derived from three independent biological replicates. Significant differences determined using Student’s *t*-test are indicated: *P<0.05.

Supplementary Material 2: Figure S2 Transcript profiles of *TaSRLK* in susceptible wheat cultivar MX 169 plants inoculated with CYR23 (incompatible interaction) or CYR31 (compatible interaction). The *TaEF-1α* gene was used as the reference. The transcript level of *TaSRLK* in the leaves infected with CYR23 at 0 hpi was standardized as 1. Differences were assessed using Student’s *t*-test (*P <0.05).

## Data Availability

All data and materials are available in the paper and online supplemental files.

## Abbreviations

*Pst*: *Puccinia striiformis* f. sp. *tritici*
LecRLKs: Lectin receptor-like kinases
ROS: Reactive oxygen species
PTI: PAMP-triggered immunity
ETI: Effector-triggered immunity
PRRs: Pattern recognition receptors
PAMPs: Pathogen-associated molecular patterns
LRR-RLK: Leucine-rich receptor-like kinase
FLS2: Flagellin-sensitive 2
flg22: Flagellin protein
Xoo: *Xanthomonas oryzae *pv.* oryzae*
AtCERK1: *Arabidopsis* *thaliana* chitin elicitor receptor kinase 1
TM: Transmembrane
PcEXLX1: *Phytophthora capsica* Expansin-like protein
BAK1: Brassinosteroid receptor-associated kinase-1
dpi: Days post-inoculation
WGA: Wheat germ agglutinin
DAB: 3,3’-diaminobenzidine
N. benthamiana: *Nicotiana benthamiana*
T. aestivum: *Triticum aestivum*
PVX: Potato virus X
BAX: Proapoptotic protein
TaPrx1: Wheat peroxisome1 like
Co-IP: Co-immunoprecipitation
EV: Empty vector
SP: Signal peptide
B-lectin: Bulb-lectin domain
SLG: S-locus glycoprotein
PAN: Plasminogen-applenematode
STK: Serine/threonine kinase
SA: Salicylic acid
JA: Jasmonic acid
