# Supplementary figures and images for: A G-type lectin receptor-like kinase TaSRLK confers wheat resistance to stripe rust by regulating the reactive oxygen species signaling pathway
Source: Stress Biol. 2025 May 23;5(1):37. doi: 10.1007/s44154-025-00225-w (PMC12102409; doi:10.1007/s44154-025-00225-w)

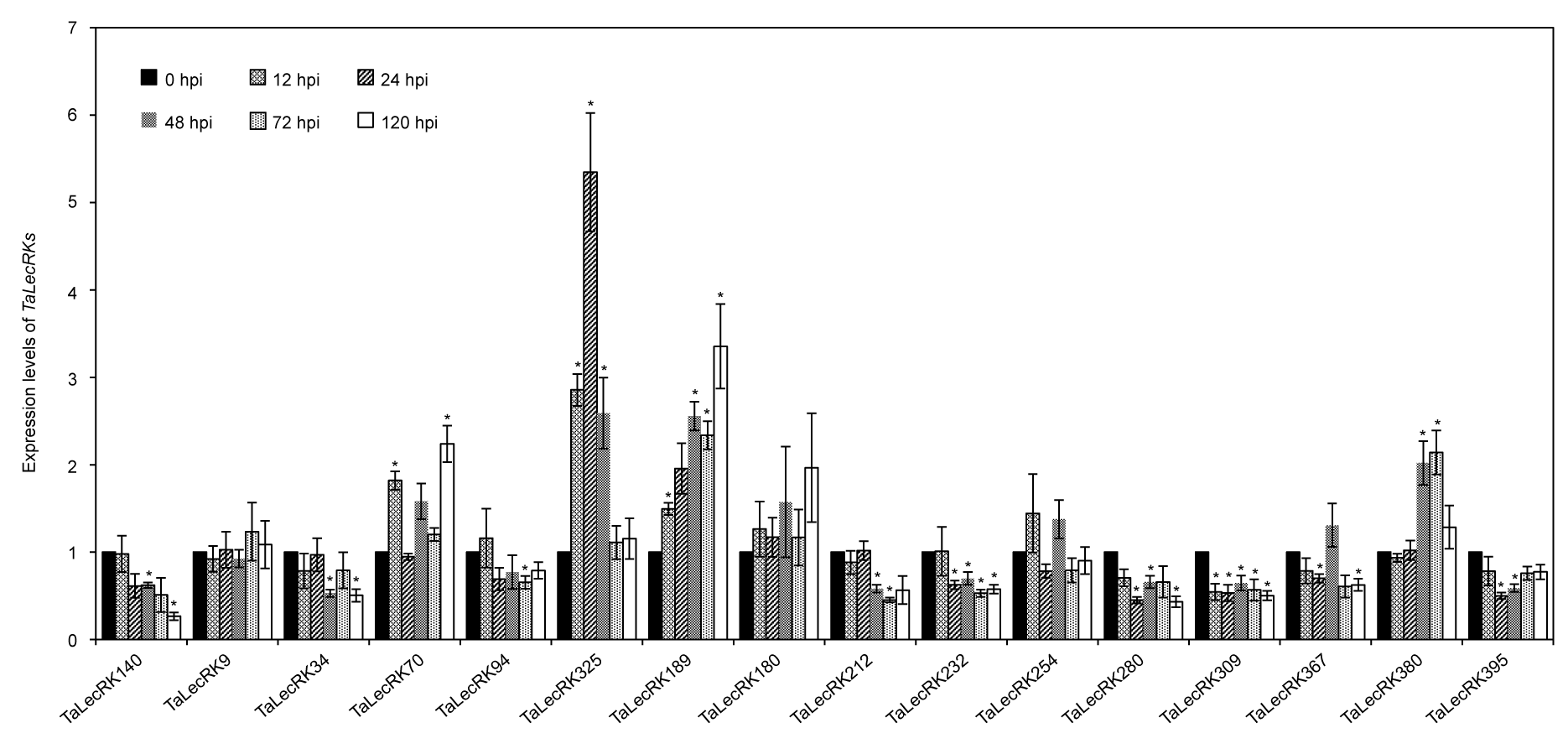

Supplement: Supplementary file 1 — Supplementary Material 1: Figure S1 Expression pattern of 16 G-type TaLecRKs in response to Pst infection. Suwon11 leaves were inoculated with Pst race CYR23. RNA was extracted from the sampled leaves at 0, 12, 24, 48, 72 and 120 hours post inoculation (hpi). The 2−ΔΔCT method was used for relative expression analysis. Wheat TaEF-1α was used as internal controls for normalization. To facilitate comparisons, the transcript levels of each TaLecRK at 0 hpi with CYR23 were set to 1. The error bars in the data represent the standard error derived from three independent biological replicates. Significant differences determined using Student’s t-test are indicated: *P<0.05. [file 44154_2025_225_MOESM1_ESM.tif]

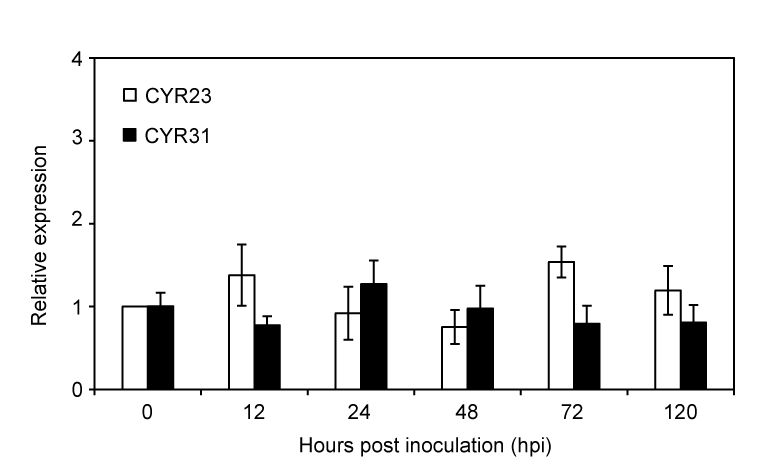

Supplement: Supplementary file 2 — Supplementary Material 2: Figure S2 Transcript profiles of TaSRLK in susceptible wheat cultivar MX 169 plants inoculated with CYR23 (incompatible interaction) or CYR31 (compatible interaction). The TaEF-1α gene was used as the reference. The transcript level of TaSRLK in the leaves infected with CYR23 at 0 hpi was standardized as 1. Differences were assessed using Student’s t-test (*P <0.05). [file 44154_2025_225_MOESM2_ESM.tif]
